# The Determinants of Market Participation and Its Effect on Food Security of the Rural Smallholder Farmers in Limpopo and Mpumalanga Provinces, South Africa

**DOI:** 10.3390/agriculture12071072

**Published:** 2022-07-21

**Authors:** Simphiwe Innocentia Hlatshwayo, Temitope Oluwaseun Ojo, Albert Thembinkosi Modi, Tafadzwanashe Mabhaudhi, Rob Slotow, Mjabuliseni Simon Cloapas Ngidi

**Affiliations:** 1 African Centre for Food Security, School of Agricultural, Earth and Environmental Sciences, College of Agriculture, Engineering and Science, University of KwaZulu-Natal, Private Bag X01, Scottsville, Pietermaritzburg 3201, South Africa; 2 Centre for Transformative Agricultural and Food Systems, School of Agricultural, Earth and Environmental Sciences, College of Agriculture, Engineering and Science, University of KwaZulu-Natal, Private Bag X01, Scottsville, Pietermaritzburg 3201, South Africa; 3 Department of Agricultural Economics, Obafemi Awolowo University, Ile-Ife 22005, Nigeria; 4 Disaster Management Training and Education Centre for Africa, University of the Free State, Bloemfontein 9301, South Africa; 5 International Water Management Institute (IWMI), Southern Africa, Pretoria 0184, South Africa; 6 Centre for Transformative Agricultural and Food Systems, School of Life Sciences, College of Agriculture, Engineering and Science, University of KwaZulu-Natal, Private Bag X01, Scottsville, Pietermaritzburg 3201, South Africa; 7 Department of Agricultural Extension and Rural Resource Management, School of Agricultural, Earth and Environmental Sciences, College of Agriculture, Engineering and Science, University of KwaZulu-Natal, Private Bag X01, Scottsville, Pietermaritzburg 3201, South Africa

**Keywords:** food in security, smallholder farmers, market participation, extended ordered probit regression model

## Abstract

Addressing the disproportionate burden of food insecurity in South Africa requires targeted efforts to help smallholder farmers to access markets. The purpose of this study was to assess determinants of market participation and its contribution to household food security. The secondary data used in this study were collected from 1520 respondents; however, 389 smallholder farmers participated in the market. The Household Food Insecurity Access Scale revealed that out of the total sample size, 85% of the households were food insecure while 15% were food secure. Gender of household head, receiving social grants and higher wealth index positively impacted market participation. Having a family member with HIV had a negative impact on market participation among smallholder farmers. The results from the extended ordered probit regression model showed that household size, having a family member with HIV and agricultural assistance had a positive and significant contribution to the household food insecurity situation of the smallholder farmers. On the other hand, the educational level of household head, ownership of livestock, age of household head, gender of household head, and having access to social grants had a negative and significant effect on the food insecurity status. Access to education and the market can improve household food security. Linking smallholder farmers, particularly women and aged farmers, to markets should form an intrinsic part of the government’s efforts to improve farming and food security and increase access to diversified food.

## 1 Introduction

Food insecurity is still a major concern worldwide, and the chances of achieving the Zero Hunger target by 2030 are slim, as more than 820 million people are experiencing hunger and malnutrition [1]. Most food-insecure and malnourished people are found in developing regions, mostly in sub-Saharan Africa [2–4]. While South Africa is considered food secure as a nation, not all South Africans are considered food-secure at the household level [2]. For instance, about 9.34 million households (16% of the population) in South Africa will face severe food security levels in 2020 [5]. About 20.6% of households will experience hunger in 2020 [6]. Household food security is highly dependent on income as most households rely on purchased food. However, about 55.5% of South African residents live in poverty, the majority being children, women, and the elderly [7].

Additionally, 25.2% of South Africans live below the food poverty line (FPL) [8]. The country is facing epidemiological and nutritional transformation (about 25% of children under the age of 5 years are stunted, and 40% of women are obese) [8,9]. Approximately 80% of South Africa’s rural population attain their livelihood from agriculture [10]. This population generally depends on smallholder agriculture for food, employment, and income [10]. This shows that agriculture remains a backbone in many rural households, vital in improving food security and reducing poverty [2]. Despite all the potential that smallholder agriculture has, the sector is still faced with several challenges that limit its potential to ensure that all people in the rural areas can acquire sufficient quantity and quality of food, either through their own production, purchase, or equitable food distribution [11–13]. Smallholder farmers that operate under smallholder agriculture can be identified as those who own small areas of land (less than 2 ha), on which they produce crops and rear livestock with limited resources [14]. South Africa has approximately two million smallholder farmers [2]. These farmers are mainly involved in subsistence farming, producing mostly for their own consumption and selling the excess within their local areas. Smallholder agriculture is categorized by low productivity, poor infrastructure, low input, lack of capital, technology and knowledge, subsistence production system, inability to reach economies of scale which are important to compete in the regional and global markets and inaccessibility to input and output markets [15–17]. These constraints are coupled with the increase in population growth that pressures the sector in generating enough food for the South African population. However, South Africa has a great potential for agriculture, so promoting market-oriented agriculture would make a remarkable impact to enhancing rural farm households’ well-being in terms of food security. Market participation among smallholder farmers is expected to lead to more specialized production systems that will ensure the efficient use of resources [18].

Smallholder farmers’ livelihoods in developing countries can be improved by integrating them into the market [19]. Market entrance is a strategy that can ensure that smallholder farmers’ food necessities are met and that they make adequate income for their immediate consumption needs, investments and social purposes [20]. It can also lead to more comparative advantages in resource use which can be shown in improved productivity through economies of scale, higher incomes, and access to new opportunities, which can lead to well-being gains for smallholder farmers [20,21]. Smallholder farmers can be consumers and producers in the market. They can participate in the agricultural output markets and derive income from sales, which can be used to buy food items not available from their own production, thereby contributing to their dietary diversity and food and nutrition security [22]. Consequently, market participation is expected to affect several aspects of rural households that influence their well-being, such as income, productivity, production, and food and nutrition security. Despite the potential benefits that market access can offer, smallholder farmers may still not interact directly with the market. Smallholder farmers’ market participation is affected by many factors, such as market imperfections, technical inability, inappropriate agricultural policies, limited knowledge, price instability, and socio-economic factors [23,24]. This has resulted in smallholder farmers producing mainly starchy cereal crops and few protein-enriched crops, limiting food diversity from their own production [25]. The failure of smallholder farmers in accessing markets has shown that there are inequalities in the food security strategy implemented by the South African government [26]. The National Food and Nutrition Security Policy was developed in 2013 to ensure the accessibility, availability and affordability of safe and nutritious food at household and national levels [27]. However, there is still an issue of availability, affordability and accessibilities within smallholder production [28]. It is, therefore, important to assess how market participation affects food and nutrition, so that evidence-based information can be provided to improve food security and market policies.

Despite the importance of market participation in the food security strategies of many developing countries such as South Africa, limited empirical knowledge exists on the linkages between the two. As posited by Fusco et al. [29], similar observations were found in developed countries, suggesting a need to give attention to developing and developed countries. Other studies [30–32] have also investigated the problem of food security in economically developed countries. Several studies [33–36] have paid more attention to analysing factors determining farmers’ market participation in various parts of developing countries. On the other hand, food security studies [12,37–39] have not investigated the role of market participation. There is, therefore, a need for quantitative research linking market participation to food security indicators to offer empirical-based evidence of the role market access plays in reducing rural hunger, food insecurity, and malnutrition. Against this backdrop, the study here presented has the following aims: (1) to determine the factors that influence market participation among smallholder farmers, and (2) to quantify the effects of market participation on rural farming households’ food security in two Provinces of South Africa.

## 2 Materials and Methods

### 2.1 Description of Study Areas

**Figure 1:**
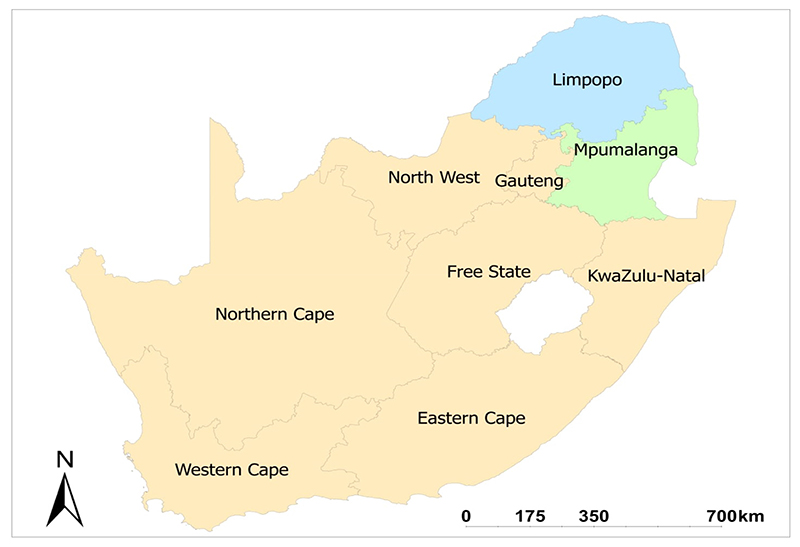
A map of South Africa showing the two provinces (Mpumalanga and Limpopo) used in this study. Source: http://www.demarcation.org.za/ accessed on 1 July 2022.

The study used secondary data collected in the nine provinces of South Africa. The data was collected in the 2016/2017 season to acquire all the necessary information on the livelihood systems of rural households and assess the extent of nutrition and food insecurity in rural communities of South Africa (available at: www.drdlr.gov.za accessed on 20 June 2020). The current study focused on two provinces (Limpopo and Mpumalanga) (as shown in Figure 1), with many smallholder farmers. According to Lehohla [40], about 68% of the provinces’ land area is utilized for agricultural intentions. The agricultural sector combines emerging crop, subsistence, and commercialized and livestock farming [41]. The Mpumalanga province is separated into Lowveld and Highveld districts and has an extremely diverse climate [42]. The Lowveld areas have a subtropical climate and mild winters, while the Highveld receives extremely cold frosty winters with temperate summers [43]. The temperature averages 17.6 °C and 63.7 °C. Rainfall varies between 750 and 867 mm p.a. [44]. The distribution of rains in Limpopo province is erratic and inconsistent. During summer (October–March), the typical rainfall is ±500 mm p.a., whereas the remaining three seasons are normally dry [45]. The province can receive extremely high temperatures in summer that range between 45 °C and 50 °C; however, the average summer temperature is about 27 °C. These extreme climatic conditions result in persistent droughts [45]. The major crops grown by smallholder farmers in the two provinces include maize, potatoes, beans and vegetables [40].

### 2.2 Data Collection Method

The study used a quantitative research method to collect data. The multi-stage stratified random sampling technique was used to select a sample size randomly. The quantitative information was collected using a survey on four districts of Mpumalanga and three districts of Limpopo; the required data (refer to Table 1) was on key indicators of agriculture, nutrition and food security. The sampled population in each site was divided into different strata considering characteristics such as institutional, technical and socio-demographic factors. A total sample size of 1520 was used from two selected provinces (Mpumalanga and Limpopo). The secondary data were obtained from the Department of Agriculture, Land Reform, and Rural Development (DALRRD), and the SAVAC collected it in 2016/2017.

So far, there is minimal, rather than no, other broad food and nutrition security dataset collected in the country at a household level. Furthermore, the recommendations presented in this study suggest that it would benefit the government, as a custodian of these data, to address issues that arise from existing policies and extension programmes. Permission to use this dataset was granted by DALRRD.

### 2.3 Data Analysis

The quantitative data were analyzed using STATA statistical software (version 13) and Statistical Software for Social Sciences (SPSS) version 24. The descriptive statistics analysis was performed to compare the sampled population’s socio-economic factors and food security status between smallholder farmers who participated in the market and those who did not. The food security assessment used the internationally accepted food measurement tool: The Household Food Insecurity Access Scale (HFIAS).

The HFIAS was used to evaluate the “access component of household food insecurity” considering the information provided in a month [46]. This scale has about nine questions based on an individual’s food access uncertainty and anxiety. Also, the questions were based on the amount of quality food consumed by a household. Tables 2 and 3 show the responses received from participants when they were asked the nine questions. The main aim of the survey was to evaluate whether participants had encountered any problems accessing food for 30 days. The questions that were asked were divided into three parts which showed an increasing level of severity of food insecurity: (question 1), inadequate quality (questions 2–4) and insufficient intake (questions 5–9). The participants were asked to specify the occurrence of the situation, i.e., if the situation had occurred rarely or never occurred (once or twice in the past month), sometimes (three to ten times in the past month) or often (more than ten times in the past month).

The study assessed whether market participation by smallholder farmers would increase their food security. It was hypothesised that smallholder farmers participating in the market could experience improved food security. The income obtained from their produce could be used to buy other healthy foodstuffs they cannot produce and to buy more inputs for sustainable production and improved productivity.

#### Extended Ordered Probit Regression Model for Ordered Responses

The extended ordered probit regression model measured food insecurity severities among smallholder farmers. In this study, food insecurity severity was considered an ordered response, since the ordered probit regression models deal with the indexed nature of different response variables. Underlying the indexing in such models is a latent but continuous descriptor of the response. In an ordered probit model, the random error related to this continuous descriptor is expected to take a normal distribution. The ordered probit regression model is preferred to multinomial logit and other probit models as it ordinarily allows the data and increases the degrees of freedom available for estimating parameters.

The ordered probit can be estimated via several commercially available software packages and is theoretically superior to most other models for the data analyzed in this work. The following specification for the extended ordered probit regression model was used:

$$T_{n}^{*}=\beta^{\prime }Z_{n}+\varepsilon_{n}$$

where $T_{n}^{*}$ is the latent and continuous measure of food insecurity severity faced by smallholder farmers *n*, *Z_n_* is a vector of explanatory variables describing the socio characteristics of farmers, *β* is a vector of parameters to be estimated, and *ε_n_* is a random error term (assumed to follow a standard normal distribution).

The observed and coded discrete food insecurity severity variable, *T_n_* is determined from the model as follows:

$$\begin{array}{ll} T_{n}= & 0\;\text{if}\;-\infty \leq T_{n}^{*}\leq \mu_{1}(\text{Food secured}\;)\\ & 1\;\text{if}\;\mu_{1}<T_{n}^{*}\leq \mu_{2}\text{(Mildly to food secured)}\;\\ & 2\;\text{if}\;\mu_{2}<T_{n}^{*}\leq \mu_{3}\text{(moderate to food insecured)}\;\\ & 3\;\text{if}\;\mu_{3}<T_{n}^{*}\leq \infty \text{(Severely food insecured)}\; \end{array}$$

where the *μ_i_* represent thresholds to be estimated (along with the parameter vector *β*).

The probabilities associated with the coded responses of an ordered probit model are as follows:

$$\begin{array}{ll} P_{n}(0) & =\mathrm{Pr}(T_{n}=0)=\mathrm{Pr}(T_{n}^{*}\leq \mu_{1})=\mathrm{Pr}(\beta^{\prime }z_{n}+\varepsilon_{n}\leq \mu_{1})\\ & =\mathrm{Pr}(\varepsilon_{n}\leq \mu_{1}-\beta^{\prime }z_{n})=\phi (\mu_{1}-\beta^{\prime }z_{n})\\ P_{n}(1) & =\mathrm{Pr}(T_{n}=1)=\mathrm{Pr}(\mu_{1}<T_{n}^{*}\leq \mu_{2})\\ & =\mathrm{Pr}(\varepsilon_{n}\leq \mu_{2}-\beta^{\prime }z_{n})-\mathrm{Pr}(\varepsilon_{n}\leq \mu_{1}-\beta^{\prime }z_{n})\\ & =\phi (\mu_{2}-\beta^{\prime }z_{n})-\phi (\mu_{1}-\beta^{\prime }z_{n})\\ P_{n}(k) & =\mathrm{Pr}(T_{n}=k)=\mathrm{Pr}(\mu_{k}<T_{n}^{*}\leq \mu_{k}+1)\\ & =\phi (\mu_{k+1}-\beta^{\prime }z_{n})-\phi (\mu_{k}-\beta^{\prime }z_{n})\\ P_{n}(K) & =\mathrm{Pr}(T_{n}=K)=\mathrm{Pr}(\mu_{K}<T_{n}^{*})\\ & =1-\phi (\mu_{K}-\beta^{\prime }z_{n}) \end{array}$$

where *n* is an individual, *k* is a response alternative, *P*(*T_n_* = *k*) is the probability that individual *n* responds in manner *k*, and *ϕ* () is the standard normal cumulative distribution function. In the increasing nature of the ordered classes, the interpretation of this model’s primary parameter set, *β* is as follows: positive signs indicate higher food insecurity severity as the value of the associated variables increase, while negative signs suggest the converse. These interactions must be compared to the ranges between the various thresholds to determine the most likely food insecurity classification for a particular smallholder farmer.

## 3 Results

### 3.1 Descriptive Analysis of the Results

The data reveals that out of the total sample of 1520 smallholder farmers, 389 (representing 12.6%) of the smallholder farmers were market participants, while 1131 (representing 74.4%) had not participated in the market, as shown in Table 4.

Tables 5 and 6 show the summary statistics of the explanatory variables used in this study. Table 5 indicates the different means and standard deviations of smallholder farmers’ demographic characteristics. The results showed that the mean household age was 49.12 years. The mean household size was 4.93. The mean total output of crops was 3556.22 kg.

Table 5 shows the differences in explanatory factors between market participants and non-market participants. The results showed that only 26% had access to agricultural assistance among market participants, while 74% did not. About 72% of the non-market participants did not have access to agricultural access, whereas 28% did have access. The results also revealed among market participants, 23% were males, and 77% were females. On the other hand, 39% of males and 61% of females were non-market participants. Regarding livestock ownership, 37% of non-market participants owned livestock, while 63% did not have any livestock. Regarding market participants, 23% had livestock, whereas 77%did not own any. The results also showed that about 34% of non-market participants had access to market information, while 66% did not have access. Among market participants, only 15% had access to market information, while 85% did not have access.

#### 3.1.1 Occurrence of Food Insecurity by Household Characteristics Based on HFIAS Categories

The Household Food Insecurity Access Scale, whichis aimed at determining households’ access to food, revealed that overall (1520 sample size), 85% of the households were fo od insecure, and only 15% were food secu res, indicating that the majority of the households were experiencing difficulties when it comes to food access. Regarding the HFIAS tool categories, 51% were either severely or moderately severely food insecure, indicating serious difficulties relating to access to food for ffiosec surveyed households.

**Figure 2:**
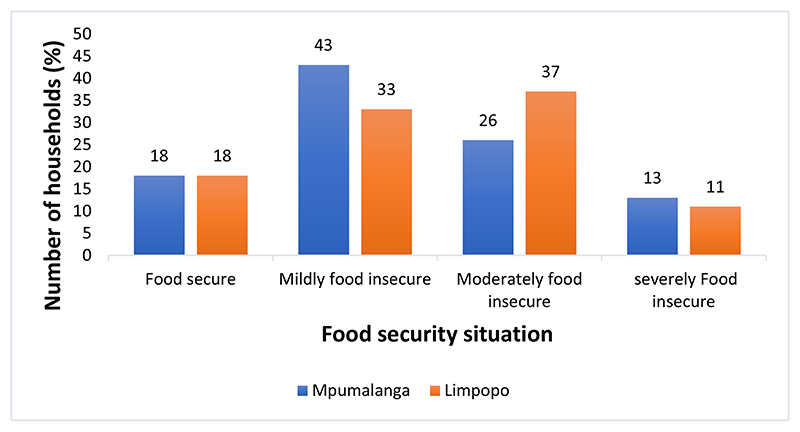
The food insecurfty situation of the smallhoMer farmers for the two provinces (Mpumalanga (*n* = 609) and Limpopo (*n* = 911)). Source: Own analysis.

Analysis of the food security situation for the two provinces revealed that the majority of farmers in Mpumalanga province were mildly food insecure (43%), while in Limpopo province, the majority of farmers were moderately food insecure (37%) (Figure 2). About 13%o f farmers were severely food ins ecure in Mpumalanga, and ab out 11% were severely food insecure in Limpopo, indicating that some of these farmers experienced difficulties accessing food.

#### 3.1.2 Determinants of Market Participation among Smallholder Farmers

The results in Table 7 show different factors that affected smallholder farmers’ market participation in the Mpumalanga and Limpopo provinces of South Africa. The marginal analysis results showed that the gender of the household head had a positive, statistically significant (*p* < 0.10) impact on market participation among smallholder farmers. This means that more males participated in the market. Having a family member with HIV had a negative and statistically significant impact on market participation among smallholder farmers.

Contrary to the expectations, social grants had a positive and statistically significant (*p* < 0.01) impact on market participation among smallholder farmers, i.e., households with social grants participated more in the market than those without social grants. The increasing wealth index had a significant positive increase in market participation among smallholder farmers.

#### 3.1.3 Determinants of Market Participation in the Severity of Food Insecurity (HFIAS)–Extended Ordered Probit Regression Model

The Ordered probit regression model was estimated for food insecurity severity in terms of HFIAS of all the smallholder farmers that had or had not participated in the market. Table 8 provides the estimated results of ordered probit models of food insecurity severity of smallholder farmers who participated and those who did not participate in the market. Since the dependent variable, HFIAS, increases with food insecurity severity, positive coefficients indicate the possibility of more severe food insecurities and negative coefficients indicate otherwise.

The age of smallholder farmers that participated in the market was statistically significant at 5%, and it had a negative coefficient, i.e., as the age of smallholder farmers increased, they experienced less food insecurity (Table 8). The household size of both market participants and non-market participants had a significant positive impact on the HFIAS, i.e., an increase in household size for both farmers that participated and that did not participate in the market resulted in an increase in food insecurity severity. The gender of the household had a significant negative effect on the HFIAS of non-market participants, but with no difference for market participants (Table 8). Surprisingly, access to agricultural assistance had a significant positive impact on the HFIAS, i.e., agricultural assistance was associated with increased food insecurity severity. The educational level of the household head had a significant negative effect on the HFIAS of the non-market participants, with better-educated households that did not participate in the market being less likely to be food insecure. Also, livestock ownership had a statistically significant negative effect on household food insecurity of non-market participants, i.e., smallholder farmers who owned livestock and did not participate in the market were less likely to experience food insecurity.

It is generally assumed that higher-income households are more likely to be food secure. Indeed, income significantly negatively affected the HFIAS of the non-market participants, meaning that family members with income were food secure. Also, access to social grants significantly negatively affected the HFIAS of the market participants, i.e., smallholder farmers who received social grants and participated in the market were less likely to be food insecure. Lastly, having a family member with HIV significantly positively impacted the HFIAS of the market participants, i.e., as HIV positive household members increased, there was a likelihood that farmers who participated in the market became more food insecure (Table 8).

##### Treatment Effects of Market Participation on the HFIAS

The main objective of this study was to assess the effect of market participation on the food security of smallholder farmers in terms of HFIAS. The Extended ordered probit regression results showed that food insecurity severity was associated with the positive coefficients received from the determinants of market participation. The study recognized that smallholder farmers’ decision as to whether to participate in the market was based on various factors, such as their productive inputs and socio-demographic characteristics, which were heterogeneous and could result in self-selection bias. Therefore, a sensitivity analysis was conducted to check the robustness of the estimated results. For the whole sampled population, the Average Treatment effect on the Treated (ATT) of the three food insecurity categories was compared with the expected average effect on the three food insecurity categories, as shown in Table 9.

These results showed that there was no major difference between the expected results and the conditional treated results. This meant that the positive coefficients of the explanatory variables were associated with an increase in food insecurity severity in terms of HFAIS, whether farmers were participants or non-participants in the market. It could be concluded that the estimated effects of market participation on food security wesre also robust, in general.

## 4 Discussion

The study’s main objective was to assess the impact of market participation on the food security of smallholder farmers in the Mpumalanga and Limpopo provinces of South Africa. The overall results on HFAIS categories showed that most (85%) households were food insecure. This is because smallholder farmers in rural areas face numerous challenges threatening their access to healthy and nutritious food. Smallholder farmers in rural areas of South Africa can grow food for a living. However, they lack the necessary resources to help them continuously meet their dietary needs through production or purchase [47,48]. In this study, the determinants of market participation were assessed, followed by assessing their impact on household food security.

Gender plays an important role in agriculture; males and females have different roles in ensuring crops are produced and marketed effectively. Rural women are an essential resource in agriculture, providing labour [49] and mainly involved in the production side [50]. Males are the ones that participate more in the market. They are mainly involved in cash crops meant to provide income than in subsistence crops grown for consumption [35]. Our results confirmed that male-headed households indeed participated more in the market. On the other hand, the negative relationship between the gender of the household and the HFIAS of non-market participants implied that female-headed households were involved in other non-farm activities that provided money for them to spend on different kinds of food and enhanced household food security. However, this result was contrary to other studies [51,52]. Taruvinga et al. [51] found that female-headed households participating in the market were food secure compared to male-headed households. Magaña-Lemus et al. [52] found that male-headed households participating in the market were more secure in food as they had enough resources and capital to improve their food security. Therefore, males and females play crucial roles, ensuring their families are well taken care of and their food security is improved [35,49,50].

Smallholder farmers’ market participation was negatively affected by having a family member with a positive HIV status. This is because having a sick family member increases stress and affects other family members’ mental and physical health [53]. This affects their decision to be involved in crop production and their decision to participate in the market [53]. National Home Sharing and Short Break Network (NHSN) (Undated) stated that having a family member with HIV is associated with time, financial costs, and physical and emotional demands, which affects education/training and work decisions. According to FAO [54], HIV results in low production and productivity as it affects most farm workers, thus reducing the total harvest that can make smallholder farmers participate in the market. Most rural households depend more on social grants for a living [55]. This study confirmed that access to social grants had a positive impact on market participation and a negative effect on the HFIAS of the market participants. This result was in line with Sinyolo et al. [56]. They found that in rural areas, there are high levels of unemployed and shortages of economic opportunities, resulting in rural households depending more on social grants for everything they do. The farmers use their social grants to purchase more inputs to use on the farm and produce more for consumption and sale. Social grants can increase many rural households’ productive and human capital capacity [56]. In contrast to these results, Sinyolo et al. [56] found that social grant-dependency had a negative impact on market participation. The study concluded that social grant-dependent households are more subsistent and they produce less marketable surplus, which could lead to susceptibility to food insecurity.

Older smallholder farmers participated more in the market because smallholder agriculture mostly involved older people [57]. Older people tend to make better decisions when it comes to farming, as most of them use their retired funds to invest in farming [57]. Therefore, they produce enough varieties of crops for home consumption and sell the surplus. Sinyolo and Mudhara [12] explained that as the age of smallholder farmers increases, more experience is gained in managing resources and social capital, which then helps farmers to improve their food security. Social capital denotes the networks, contacts and trust that allow farmers to use their resources more effectively [58].

The household size of both market participants and non-market participants positively impacted the HFIAS. This is because large households tend to only produce staple crops for their survival, not for their health [59]. Moreover, an increase in household size causes farmers to produce more for consumption and fewer sales are made from agricultural products. This result was in line with that of Martey et al. [60], who reported that large household size reduces marketable surplus that might help farmers to receive income that would help them to purchase healthy foods and be food secure.

Agricultural assistance from policymakers, government and other stakeholders is supposed to improve smallholder farmers’ production, marketing and consumption, which can lead to more production of diverse crops and improve the food security of smallholder farmers. Access to agricultural assistance can help provide improved varieties and market information that can improve farmers’ access to the market and increase their knowledge of production [35,61]. It can also help farmers with the provision of varied seeds that would help them produce diverse crops for sale and consumption [62,63]. However, in Nigeria, there was a negative relationship between agricultural extension and credit market participation among smallholder rice farmers [64]. In this study, agricultural assistance increased food insecurity. The possible explanation for this might be that sometimes smallholder farmers do not receive enough or inadequate government assistance and end up utilizing whatever resources they have to produce only staple crops [2]. Extension officers understaff the agricultural sector in South Africa, and poor training on sustainable crop production methods, such as crop diversification, means they do not provide sufficient market information or support [65]. This results in farmers relying on their traditional methods to produce staple crops [57].

The educational level of the household head increased the food security of the non-market participants. This could be attributable to the fact that household heads with higher education can better access and use information that can improve their ability to improve their households’ food security. They are also able to distinguish between healthy and non-healthy foods. The result is consistent with other studies [12,66,67]. Also, the result revealed that livestock ownership negatively impacted the HFIAS of non-market participants. This implied that smallholder farmers that owned livestock and did not participate in the market were less likely to experience food insecurity. This is because livestock ownership is a sign of wealth in most developing countries like South Africa, especially in rural areas [68]. So, households with more livestock are most likely to spend more on healthy food and are food secure. Bellemare and Barrett [69] reported that livestock ownership helps ensure that food is always available as it can be sold during a food shortage.

The result showed that income had a negative effect on the HFIAS of the non-market participants. This is because households with income could spend on various foods. The result was substantiated by Gebre [70], who found evidence that employed households earning income were expected to have a positive food security status. Also, Taruvinga et al. [51] found a positive association between income and food security statuses. It can be concluded that income leads to high demand for various foods that lead to food security.

## 5 Conclusions and Recommendations

Improved education among smallholder farmers can improve both market participation and food security. Workshops and focused training that would help farmers engage with different people and encourage them to explore different things are needed. This will help in utilizing resources, as farmers will be exposed to different kinds of help and be willing to take risks. While agricultural services are expected to improve market participation and food security, the findings of this study indicated that agricultural assistance did not improve food security. There is a need to urgently address the shortage of extension officers, while also providing adequate training for improved quality service delivery to smallholder farmers. In the same vein, the age of the household showed a positive impact on food security. It is recommended that young people are also encouraged to participate in agriculture. This can be done by conducting workshops in rural areas that would demonstrate different careers in agriculture and the importance of youth involvement in agriculture.

Access to social grants showed potential in improving market participation and food security. However, some studies found social grants to be a disincentive to participate in crop production. To ensure that social grants are used effectively and sustainably, the government should reconsider the idea of giving cash to households. Sinyolo and Mudhara [56] recommended a policy option where some of the grants are offered as ‘in-kind support’, which is specific to the intended individual beneficiary, instead of fungible cash. Mtyingizane and Masuku [71] recommended that the state and development agencies consider supplementing social grant support with more sustainable food security programmes, such as investing in education and agricultural infrastructure for domestic food production. With these programmes, households would be self-reliant with sustainable means of accessing adequate food, diversified diets and benefitting from an increase in the number of daily meals.

Overall, it is advisable that the government and policymakers revise their agricultural marketing and food security policies and redo them so that they can cater to food and nutrition security improvements at household level and also consider the conditions under which smallholder farmers live and operate. The government needs to follow up on policy implementation, so that the food and nutrition status security of rural households can be improved and sustainable crop production can be attained, which would lead to more access to markets and crop sales.

## Figures and Tables

**Table 1:** A priori expectations for the explanatory variables used in the models.

Variables Names	Variable Type and Measurement	Hypothesized Effect on Market Participation	Hypothesized Effect on Household Food Security
Age of the household head	Age of the respondent head in years	±	±
Gender of household head	1 = if resp ondent is male, 0 otherwise	+	+
Marital status	1 = if the respondent is married, 0 otherwise	±	±
Household size	The farm household’s total family members	–	–
Education level of the household head	Years of education (continuous)	+	+
Ownership of livestock	1 = if the respondent owned livestock, 0 otherwise	±	±
Access to market information	1 = if respondents had received information on the market, 0 otherwise	+	+
Involvement in crop production	1 = if respondents had been involved in crop production, 0 otherwise	+	+
Disability in the family	1 = if there is a member in the family that leaves with a disability, 0 otherwise	–	–
Access to agricultural assistance	1 = if respondents had access to extension services, 0 otherwise	+	+
Family member with HIV	1 = if there is a member in the family that is HIV positive, 0 otherwise	–	–
Family member worked on a farm	1 = if there is a member in that worked on a farm, 0 otherwise	+	+
Income	1 = if there is a member in that worked for a woge salary, 0 other wise	+	+
Social grant	1 = if there is a member in a family that received a social grant, 0 otherwise	±	±
Irrigation type	1 = if the respondent had access to an irrigation system, 0 otherwise	±	±

Source: own analysis.

**Table 2:** The number of smallholder farmer respondents to each Household Food Insecurity Access Scale survey question option for the 2016/17 season in Mpumalanga.

Do You or Your Household Members Have the Following Problems with Ensuring Food Security Due to Financial Problems/Lack of Resources:	Last 30 Days			
	No	Rarely (1–2 Times)	Sometimes (3–10 Times)	Often (More than 10 Times)
Worry about not having enough food	147	212	199	51
Do not eat your kinds of preferred food	110	225	216	58
Limit the diversity/quality of meals	102	230	211	66
Consume some foods that you did not want to eat	105	227	209	67
Limit eaten food portions	161	227	179	41
Limit the number of meals	186	213	161	49
No food to eat of any kind in your household	353	139	95	21
Go to sleep at night hungry	465	85	37	22
Go a whole day and night without eating anything	496	60	30	20

Source: Own analysis.

**Table 3:** The number of smallholder farmer respondents to each Household Food Insecurity Access Scale survey question option for the 2016/17 season in Limpopo.

Do You or Your Household Members Have the Following Problems with Ensuring Food Security Due to Financial Problems/Lack of Resources:	Last 30 Days			
	No	Rarely (1–2 Times)	Sometimes (3–10 Times)	Often (more than 10 Times)
Worry about not having enough food	231	276	305	98
Do not eat your kinds of preferred food	166	286	332	123
Limit the diversity/quality of meals	157	287	329	137
Consume some foods that you did not want to eat	157	285	325	141
Limit eaten food portions	240	288	287	94
Limit the number of meals	266	268	270	104
No food to eat of any kind in your household	511	191	159	48
Go to sleep at night hungry	667	123	72	48
Go a whole day and night without eating anything	728	86	63	31

Source: Own analysis.

**Table 4:** Socio-demographic factors of smallholder farmers in Mpumalanga and Limpopo province, South Africa.

.		Market Participants	Non-Market Participants	Total
Province name	Mpumalanga	176	433	609
	Limpopo	213	698	911
Total		389	1131	1520

Source: own analysis.

**Table 5:** Demographic characteristics of smallholder farmers in Limpopo and Mpumalanga provinces, South Africa.

Variable	*Mean* ± Standard Deviation (SD)
Gender of household head	1.27 ± 0.45
Household age	49.12 ± 11.89
Marital status	4.21 ± 2.44
Household size	4.93 ± 2.71
Educational level of household	33.58 ± 40.30
Ownership Livestock	1.77 ± 0.42
Distance to the market	1.86 ± 1.82
Access to market information	1.94 ± 0.24
Access to agricultural assistance	1.92 ± 0.27
Family member with HIV	0.47 ± 0.79
Family member worked on a farm	0.98 ± 0.76
Social grant	1.99 ± 0.73
Irrigation type	1.52 ± 0.50
Total output of crops (KG)	3556.22 ± 88,187.067

Source: Authors’ own analysis.

**Table 6:** Demographic characteristics of smallholder farmers in Limpopo and Mpumalanga provinces, South Africa.

Variable	Market Participant (*n* = 389)		Non-Market Participant (*n* = 1131)		Overall Freq
	%	Freq	%	Freq	
Gender of Household					
Female	77	300	61	688	988
Male	23	89	39	443	532
Access to Agricultural Assistance					
Yes	26	100	28	318	418
No	74	289	72	813	1102
Access to Market Information					
Yes	15	60	34	387	447
No	85	329	66	744	1073
Ownership of Livestock					
Yes	23	89	37	414	503
No	77	300	63	717	1017

Source: Authors’ own analysis.

**Table 7:** Factors influencing market participation among smallholder farmers.

Market Participation	Probit			Marginal Effect		
	Coeff	St.Err.	p-Value	dy/dx	St.Err.	p-Value
Household size	0.032	0.045	0.476	0.001	0.001	0.477
Gender of household head (male = 1, 0 otherwise)	0.644	0.319	0.043 **	0.015	0.008	0.053 *
Age of household head	–0.004	0.008	0.599	–0.000	0.000	0.600
Educational level of household head	–0.258	0.426	0.546	–0.006	0.010	0.545
Marital status of household head (married = 1, 0 otherwise)	–0.151	0.452	0.739	–0.004	0.011	0.739
Agricultural assistance	0.235	0.423	0.566	–0.002	0.011	0.543
Family member with HIV	–1.222	0.473	0.010 **	–0.029	0.011	0.011 **
Social grant	1.184	0.335	0.000 ***	0.028	0.008	0.001 ***
Wealth index	1.021	0.163	0.000 ***	0.024	0.005	0.000 ***
Amount Harvested	0.000	0.001	0.785	–0.000	0.000	0.785
Constant	0.509	0.798	0.524			
Mean dependent var	0.649					
Pseudo r-squared	0.926					
Chi-square	1268					
Akaike crit. (AIC)	120.8					
Prob > chi2	0.001					
Bayesian crit. (BIC)	170.4					

Notes: Dependent variable is market participation; ***, **, * Indicate significance at 1%, 5%, and 10% level, respectively. Source: Own analysis.

**Table 8:** Determinants of household food insecurity access scale using Extended ordered probit regression.

	Non-Market Participants			Market Participants		
Household Food Insecurity Access Scale (HFIAS) Category	Coef.	Std.Err.	*p*-Value	Coef.	Std.Err.	*p*-Value
Age of household head	0.003	0.003	0.339	–0.006	0.002	0.012 **
Household size	0.039	0.019	0.000 ***	0.085	0.015	0.000 ***
Gender of household head (male = 1,0 otherwise)	–0.556	0.196	0.004 ***	–0.257	0.184	0.162
Educational level of household head	–2.301	0.640	0.000 ***	–0.637	0.548	0.245
Marital status (married = 1, 0 otherwise)	–0.664	0.944	0.482	0.700	0.629	0.266
Irrigation type	0.174	0.267	0.514	0.414	0.307	0.177
Agricultural assistance	0.134	0.201	0.000 ***	0.195	0.131	0.000 ***
Ownership of livestock	–0.785	0.404	0.052 *	–0.658	0.608	0.279
Income	–0.613	0.351	0.081 *	0.330	0.408	0.419
Social grants	0.079	0.249	0.750	–0.419	0.233	0.072 *
Wealth index	0.040	0.240	0.867	0.507	0.281	0.701
Access to market information	0.313	0.255	0.219	0.134	0.147	0.364
Disability in the family	0.574	0.950	0.546	–0.923	0.740	0.212
Family member with HIV	0.209	0.462	0.651	1.057	0.458	0.021 **
Constant	2.519	0.261	0.000			
HFIAS categories						
Cut1	–0.921	0.645	–2.184	–1.704	0.892	–3.452
Cut2	1.489	0.650	0.215	0.771	0.893	-0.979
Correlation (market participation and HFIAS categories)	–1.000					

Notes: Dependent variable is HFIAS; ***, **, * Indicate significance at 1%, 5%, and 10% level, respectively. Source: Own analysis.

**Table 9:** Treatment effect of market participation household food insecurity access scale.

Food Categories	Mean	ATT	t-Stat	% Change
p3 (mildly to food secured)	0.0614	0.0642	87.8587 ***	100%
p2 (Moderate food insecure)	0.7544	0.7675	54.5656 ***	98%
p1 (severely food insecure)	0.1840	0.1910	0.08535 ***	98%

*** Indicate significance at 1%. Source: Own analysis.

## Data Availability

Restrictions apply to the availability of these data. Data were obtained from the Department of Agriculture, Land Reform, and Rural Development (DALRRD) and are available from the South African Vulnerability Assessment Committee (SAVAC) secretariat with the permission of the Department of Agriculture, Land Reform, and Rural Development (DALRRD).
